# A human surrogate neck for traumatic brain injury research

**DOI:** 10.3389/fbioe.2022.854405

**Published:** 2022-12-19

**Authors:** Jon Farmer, Sean Mitchell, Paul Sherratt, Yusuke Miyazaki

**Affiliations:** ^1^ Sports Technology Institute, Wolfson School of Mechanical, Manufacturing and Electrical Engineering, Loughborough University, Loughborough, United Kingdom; ^2^ Department of Systems and Control, School of Engineering, Tokyo Institute of Technology, Tokyo, Japan

**Keywords:** anthropomorphic test device (ATD), neck biomechanics, bio inspired, cervical spine, biofidelic

## Abstract

Properties of the human neck such as range and resistance to motion are considered important determinants of the kinematic response of the head pre, during and post-impact. Mechanical surrogate necks (i.e., anthropomorphic test device necks), have generally been limited to a single anatomical plane of motion and an artificially high resistance to motion. The aim of this study was to present the Loughborough University Surrogate Neck that is representative of the 50th percentile human male neck, developed for motion in and between each of the anatomical planes with inertial and flexural stiffness properties matching those of a passive elastic (i.e., negligible active tension) neck muscle state. The complex intervertebral joints were reduced to three encapsulated ball joints with appropriate locations, orientations and distributed range of motion to precisely position and orientate the head with respect to the torso at the neutral position and end range of motion. A plain bearing sub-assembly was incorporated at the C1-C2 vertebral level to permit 50% of the axial rotation with negligible resistance to motion, as exhibited by humans. Detachable elastomeric elements provided resistance to motion across each ball joint and permit any orientation of the head within the physiological range of motion of the joints. The mass of the surrogate neck (1.62 Kg) was in agreement with the typical human range and similar agreement was found for the principal moments of inertia (I_xx_ 26.8 kg cm^2^, I_yy_ 20.5 kg cm^2^ and I_zz_ 14.3 kg cm^2^). Quasi-static bending moment and dynamic torque tests characterised the surrogate neck in flexion/extension, lateral flexion and axial rotation. With respect to commercial surrogate necks, the surrogate neck presented here was in closer agreement to the reported human responses, for equivalent loading conditions. The applications of a surrogate neck that can appropriately constrain the head relative to the torso are far reaching in the areas of brain injury mechanism research, and for the development and assessment of protective equipment to reduce the risk of such injuries.

## Introduction

Traumatic brain injury (TBI) is a global concern with TBI-related disabilities affecting approximately 69 million people annually (Dewan et al., 2018). TBIs can result from a direct blow to the head or an indirect inertial response to an impact elsewhere on the body. The most common causes are falls and road traffic accidents (occupants, pedestrians or cyclists) (Tagliaferri et al., 2006). The response of the human head during and after an impact can be affected by the properties of the neck, such as range and resistance to motion (Li et al., 2018), and the risk of TBIs such as subdural hematomas and diffuse axonal injuries have been correlated with the rotational response of the head (Depreitere et al., 2006; Browne et al., 2011). The degree to which an individual is braced prior to an impact (i.e., tensed musculature) and their resistance to motion during the impact (i.e., neck stiffness) has been shown to significantly affect the angular kinematics of the skull during a backwards fall (Farmer et al., 2020). For example, the magnitudes of peak angular velocity and angular acceleration of the skull were statistically higher for the low stiffness surrogate neck compared to the high stiffness surrogate neck. It is therefore necessary for laboratory investigations concerning TBIs to consider the influence of the constraint imposed by the neck on the head’s response to an impact.

The human neck is a complex biological structure containing the weight-bearing cervical spine, 22 pairs of muscles, ligaments and other soft tissues. The primary functions of the cervical spine are to provide mobility to the head, and protection to the spinal cord, along with the rest of the spinal column. Being the most mobile region of the spine, a high degree and combination of sagittal flexion/extension (nodding), lateral flexion (left and right bending), and axial rotation (left and right twisting) motions are possible. The degree to which the complex neck musculature is activated can influence the stiffness properties of the neck. A passive (low muscle activation level) neck response allows for a high degree of ‘neutral zone’ motion, i.e., angular displacement with low resistance to motion. In particular, the neutral zone of the neck’s axial rotation response can account for 50% of the total physiological motion (Panjabi, 1992). This is of particular interest when considering that some structures of the brain have a directional response dependency, such that the risk of sustaining TBIs such as Diffuse Axonal Injury (DAI) are higher due to the constraint imposed by the structures. For example, the falx and tentorium cerebri increase strain on the cerebellum and brain stem during axial rotations (Ho et al., 2017).

Given the ethical constraints surrounding TBI research involving human participants and the limitations of cadavers (e.g., no neck musculature), it is often necessary to utilise human surrogates (i.e., anthropomorphic test devices or crash test dummies) which have been developed to represent the biomechanical response of the human neck during physical laboratory testing. These surrogates have typically been simplified to improve repeatability and durability for high energy automotive collisions; however, in some cases these simplifications have limited the surrogate’s application. Specifically, commercial surrogate necks have mainly been developed to represent motion in a single plane, for example the Hybrid III surrogate neck (H3SN), BioRID, and World-SID surrogate necks have been designed for frontal, rear, and near-side automotive impacts, respectively. The aforementioned surrogate necks are primarily designed to represent the 50th percentile male, though scaling methods have been used to also represent other demographics such as the 95th percentile. The H3SN is the most used and documented surrogate neck in existence, developed in 1972 by General Motors to investigate the risk of sustaining skull fractures during a frontal car collision. However, the H3SN neck was only validated for sagittal plane (flexion and extension) motion and is reportedly 2–3 and 10 times stiffer than the human in active and passive muscle state sagittal plane bending (flexion and extension), respectively (Doherty and Paver, 1988; Cappon et al., 2000; Friedman et al., 2009). The mod-kit THOR series (metric) surrogate neck (Ridella and Parent, 2011) is considered to be the best available automotive technology and has been developed to represent motion in all three anatomical planes with both passive and active neck musculature stiffness properties. Whilst the Thor-M responses are considered a marked improvement over the H3SN, the axial rotation response is approximately an order of magnitude stiffer than the reported computational model (Duke Adult Head and Neck Model (DAHNM)) between 0 and 10 degrees of rotation and, two-three times stiffer than the DAHNM response between 40 and 80 degrees of rotation (Luck et al., 2014).

More recently, efforts have been made to match the complex motion of the sagittal plane motion of the human neck to surrogate neck approximations. Computational models have been developed to approximate the intervertebral human motion with a two-link design that has appropriate range of motion and rotational spring stiffnesses across the joints (Fanton et al., 2018). Attempts have also been made to adapt existing surrogate necks such as the H3SN, including the removal of the biased flexion *versus* extension response (Walsh et al., 2018). Additionally, the authors of the present research adopted a similar two link mechanism to represent experimental backwards falls in judo (Farmer et al., 2020). The research showed that the magnitude of angular acceleration and angular velocity of a surrogate head, during a backwards fall, can be significantly increased when constrained by a low stiffness (passive state) surrogate neck. However, the surrogate neck was only designed for motion in the sagittal plane and therefore could not adopt complex out of plane motion. In sport, and other areas, it is unlikely that impacts resulting in TBIs, occurred in a single plane of motion. Further, it has been acknowledged that the risk of TBIs, are to some degree directional dependent (Ho et al., 2017), and therefore the constraint imposed by the neck in each of the anatomical planes, is likely to be influential on the risk factors associated with the TBIs. The development of more biofidelic surrogates for body parts is an emerging topic and significantly, a recent review of impact methods for headgear testing in sport, highlighted that ‘there is an urgent need for biofidelic neck surrogates’ that are capable of multidirectional motion (Whyte et al., 2019).

The primary aim of this research was to therefore develop a surrogate neck that could represent the motion of the human neck in and between the anatomical planes. The target stiffness response was in the order of reported osteoligamentous cervical spine and human volunteer passive state data. The further aim was to show through validation that the neck’s response was comparable to the reported human response data.

## Materials and methods

### Definition and articulation of a neutral geometric posture

The design intent was for the Loughborough University Surrogate Neck (LUSN) to be as representative of the 50th percentile male in as many aspects as possible. It is noted that the ‘average’ male that is amalgamated from various sources may not indeed represent any single individual. However, in line with previous surrogate developments, the intent here was to design a cost-effective solution that matches key average dimensions, measured from a large target demographic; whilst accepting that the result may be an ATD that exactly matches no living human. The assumption is that the ATD will respond in a human like manner and produce ‘average’ outcomes, as though the target demographic had been tested and the results averaged. This ‘representative’ ATD is then assumed to have relevance for the entire demographic and a cost, which is proportional to this greater utility.

**FIGURE 1:**
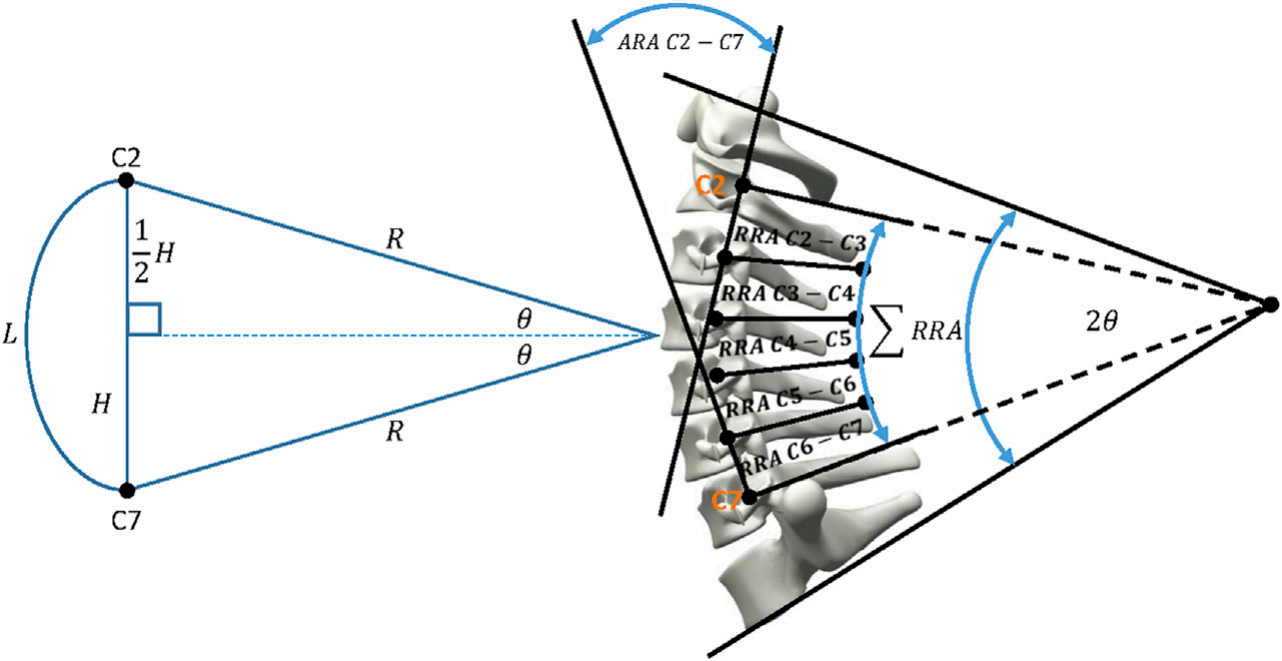
Harrison’s model of the cervical spine’s lordotic profile with the resulting geometry of the digital cervical skeletal system.

A neutral posture of the 50th percentile skeletal male (stature: 1758 mm; age: 32 years) in a standing position was defined and constructed in Siemens NX CAD software using three-dimensional cervical vertebrae models adapted from Magee (2009), as the basis. The vertebral body geometry (heights and depths) and intervertebral disc spacings were matched to mean values of the 50th percentile male (Magee, 2009). The location and orientation of the second and seventh cervical vertebrae (C2 and C7) were used as key references for defining the cervical lordosis. Harrison’s Posterior Tangent model (Harrison et al., 1996; Harrison et al., 2004), considered to be the most appropriate method of indicating the level of cervical lordosis (Magee, 2009), was used to fit a circular arc to the lordotic profile of the cervical spine. Between C2 and C7, the arc length ( L ) represents the length of the cervical spine and the chord of this arc represents the height ( H ). The height is measured as the distance between the postero-inferior body of C7 vertebra to the postero-superior body of C2 vertebra and the arc length is calculated along the posterior longitudinal ligament. The geometrical spinal model is represented in Figure 1 and is defined by the following equations Eqs 1, 2:

$$\frac{H}{L}=\frac{\mathrm{Height}}{\text{Length}}=\frac{\mathrm{Chord}}{\mathrm{Arc}\;\mathrm{length}}=\frac{C}{S}=\frac{2R\sin\left(\theta \right)}{R2\theta }=\frac{\sin\left(\theta \right)}{\theta }\;,$$ (1)

$$8x+7\left(\frac{2}{5}\right)x=2\theta ,$$ (2)

Where, H represents the height (chord) of the cervical spine, L represents the length (arc length) of the cervical spine and x represents the height of a vertebral body in degrees of the total lordotic arc. The total arc, known as the ARA, is defined by 2θ and dictates the sum of the relative rotation angles (RRA) between adjacent vertebrae. The lordotic profile of the cervical spine has a summated height of the vertebral bodies and discs of 136.5 mm. The height to length ratio of 0.97 (Harrison et al., 2004) resulted in a circle radius of 159.5 mm, upon which the posterior borders of the vertebral bodies were aligned. C2-C7 sagittal vertical analysis (SVA) was used to define the global orientation of the cervical spine such that the C2 inferior posterior corner had a relative anterior translation of 3.9 mm to the C7 superior posterior corner (Magee, 2009).

The articulation of the neutral posture of the cervical spine required the application of the intervertebral ROM at the appropriate instantaneous centres of rotation (ICR) locations. The maximum reported physiological limits of intervertebral motion for a 50th percentile male were defined by the data presented in the review of Waiter (2006) and are summarised in Table 1.

**TABLE 1:** The maximum intervertebral range of motion (degrees) for each bending mode of the cervical spine.

Segment	Flexion (°)	Extension (°)	Axial rotation (°)	Lateral flexion (°)
C0-C1	19.44	19.48	0.00	8.64
C1-C2	13.32	13.35	40.5	10.79
C2-C3	8.10	8.12	9.00	10.32
C3-C4	11.88	11.91	9.00	10.52
C4-C5	12.33	12.36	9.00	9.65
C5-C6	13.50	13.53	9.00	8.91
C6-C7	5.76	5.77	8.10	4.12
C7-T1	5.76	5.77	5.40	4.12

ICRs are an important determinant in predicting the motion path of the cervical spine and provide a quantitative location to apply an intervertebral ROM to a superior vertebra with respect to an adjacent inferior vertebra. Motion in the sagittal plane has been the major focus of previous research and as such most studies on ICRs and ROM are in this plane. To the best of the author’s knowledge no such quantitative data exists for the lateral flexion response of the cervical spine. It was therefore assumed that the ICR locations of the vertebrae in the sagittal plane could be reasonably applied for the case of lateral flexion. Normalised values of ICR locations, accounting for differences in the sizes of individual vertebral bodies, have been reported in the literature (Amevo et al., 1991; Charlton, 2015) and are presented in Table 2. The two studies report similar normalised values of ICRs for the C2/C3 to C7/T1 segmental levels. The ICR locations of the upper cervical spine have rarely been quantified. The most complete dataset was provided by Chancey et al. (2007) who reported C0-C1 and C1-C2 ICRs with respect to anatomical landmarks of the skull. Their findings agreed with Van Mameran et al. (1992). Chancey et al. (2007) also reported bony landmark positions with respect to the external auditory meatus (EAM) location. To account for likely differences in the geometry between individual skull models, the absolute ICR measurements were normalised by calculating geometrical ratios of the skull, for example the EAM to Nasion *versus* the overall head length ratio was compared. The ICR locations for the upper cervical spine are also shown in Table 2.

**TABLE 2:** The absolute reported values of upper cervical spine ICR locations with respect to the EAM (mm) and normalised lower cervical spine ICR location ratios with respect to the inferior vertebral body dimensions.

	Amevo et al. (1991)		Charlton (2015)		Chancey et al. (2007)	
Segment	Anterior x ratio	Cephalad z ratio	Anterior x ratio	Cephalad z ratio	Anterior x offset from EAM (mm)	Inferior x offset from EAM (mm)
C0/C1					−22.5	22.6
C1/C2					−7.4	46.7
C2/C3	0.30	0.35	0.27	0.36		
C3/C4	0.29	0.51	0.32	0.52		
C4/C5	0.36	0.63	0.36	0.60		
C5/C6	0.41	0.81	0.39	0.78		
C6/C7	0.49	0.99	0.44	0.95		
C7/T1	0.49	0.99	0.44	0.95		

**FIGURE 2:**
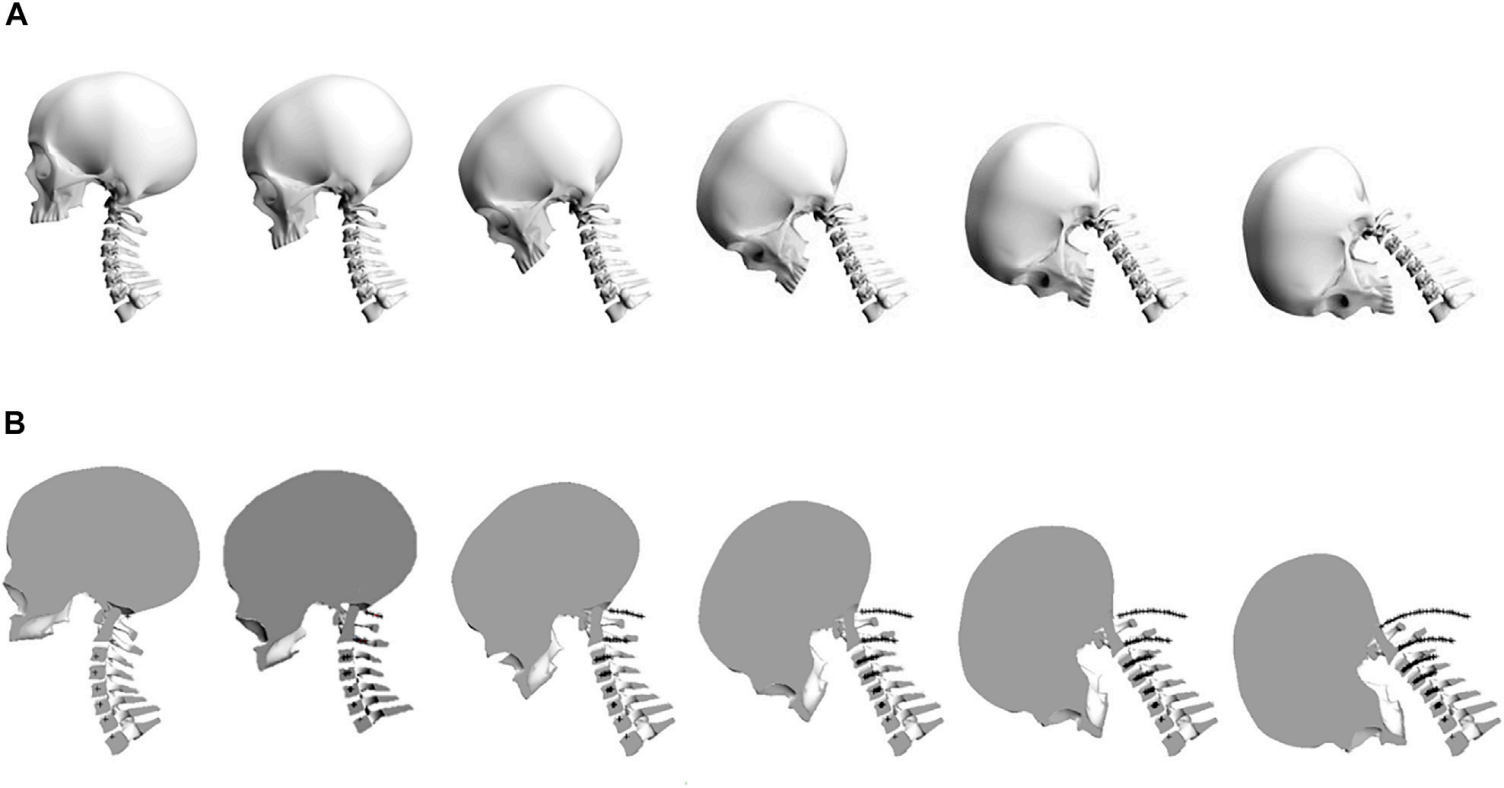
**(A)** The stepwise application of intervertebral range of motion in the flexion bending mode. **(B)** The locus of motion of the intervertebral ICR locations throughout flexion.

These intervertebral ROM and ICR location values were applied to the neutral cervical spine model in a first order mode. A proportion of the total intervertebral ROM was applied such that all vertebrae and the skull, above and inclusive of the C7 vertebra were rotated about the ICR of C7-T1 by one-fifth of its total ROM. The process was repeated for all vertebrae above and inclusive of C6 and these were rotated about the C6-C7 ICR and so forth, until the final iteration involved rotating the skull about the C0-C1 ICR. The above process was then repeated for each fraction of the total ROM until all vertebrae were positioned at their end ROM. A visual representation of the orientation and location of the cervical spine and skull in its flexion mode can be seen in Figure 2.

### Mechanical joint approximation

**FIGURE 3:**
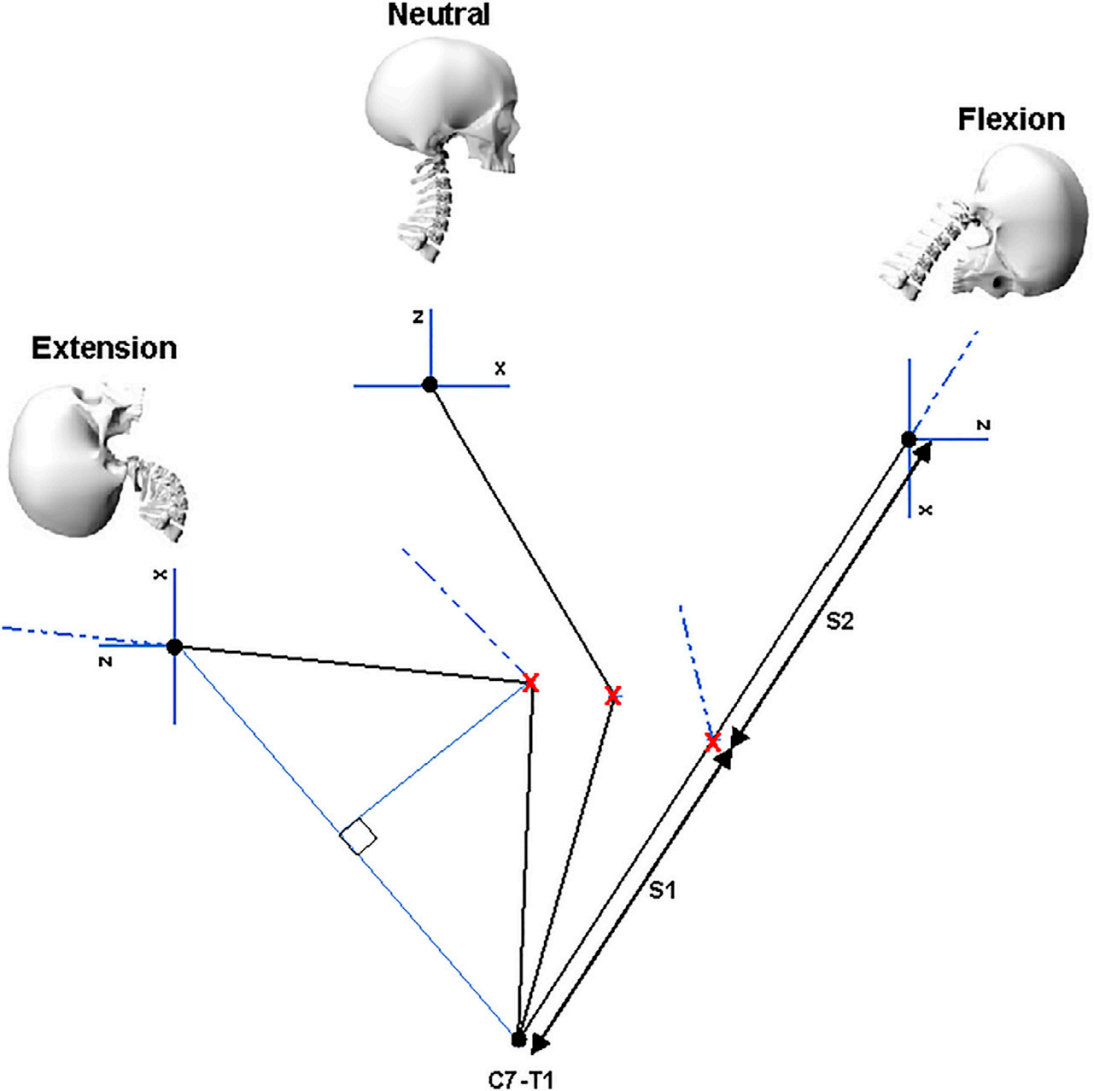
Location of the third joint centre as defined by the anterior intercept of two circles with equal radii, equidistance from the C0-C1 and C7-T1 ICR locations in the mid-sagittal plane. The resulting orientation of the Frankfort plane in the extension and flexion bending modes is also shown.

A geometrical approach was taken to inform the number and location of joints in the LUSN’s design. The point to point distance between the C7-T1 ICR and the C0-C1 ICR was considered along the mid-sagittal plane for the cervical spine in the neutral, full flexion and full extension locations. The distance between C7-T1 to C0-C1 ICRs was found to increase and decrease for flexion and extension, respectively, such that a single segment–two joint design that spanned the two ICR locations could not satisfy the loci of motion from extension to flexion. A third joint was therefore added to the model and its location was defined by the point of intersection of two circles with radii equal to half the distance between the C7-T1 ICR and the C0-C1 ICR in full flexion (Figure 3). This location is represented by a ‘**red cross**’ in Figure 3. The length of segment one (S1) and segment two (S2) are therefore equal. The resulting orientation of the head (Frankfort horizontal plane) is represented by the ‘**blue x-z axes’**, where positive x and z are towards the anterior and inferior skull, respectively.

**FIGURE 4:**
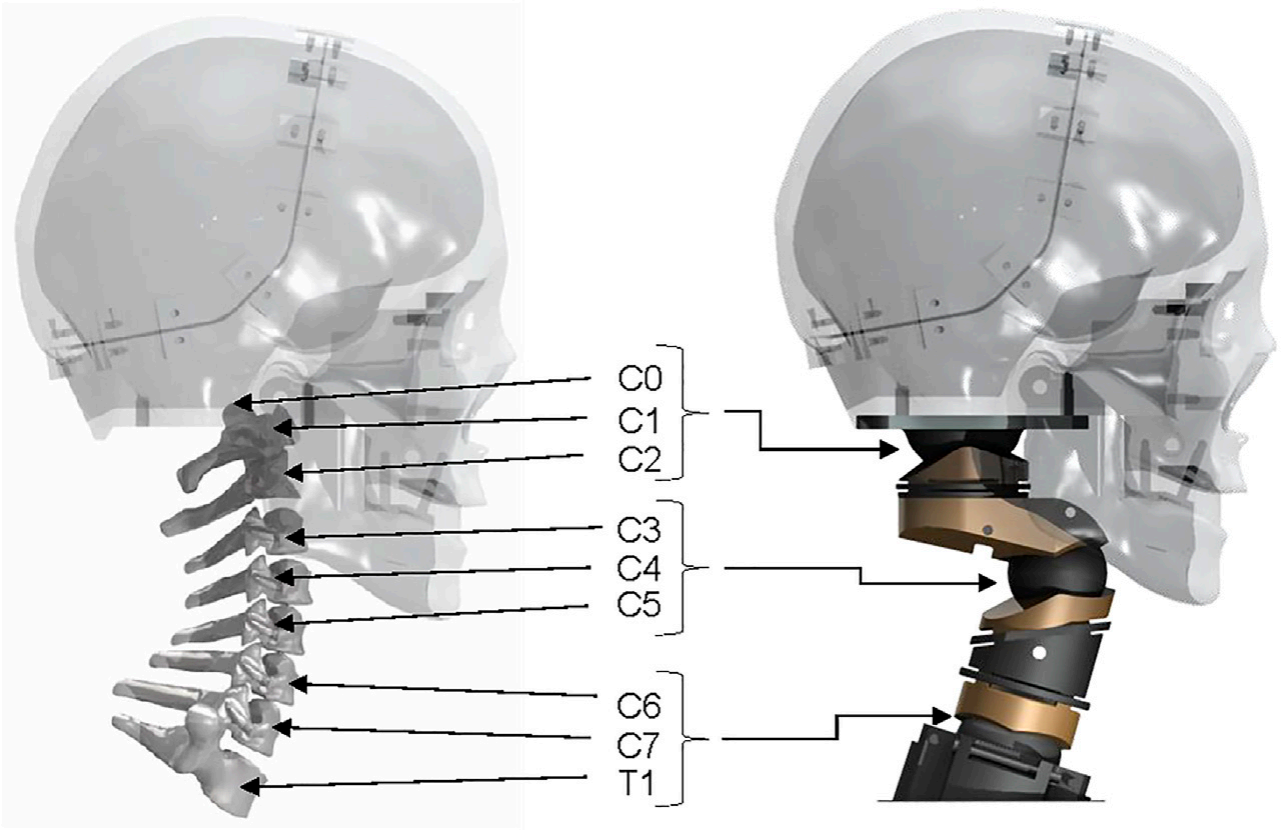
A comparison of the digital human cervical spine in combination with the Tokyo Institute of Technology surrogate and the LUSN in combination with the Tokyo Institute of Technology surrogate head.

The LUSN constitutes three encapsulated ball-type joints with superior and inferior bodies, acting to restrict ROM of each joint through contoured mating surfaces, in combination with a plain bearing axial joint to provide a high degree of axial rotation ROM. The upper assembly consists of a truncated hemispherical ball joint and plain bearing to represent the human occipito-atlanto-axial joint (C0-C2 vertebrae). The human C0-C1 joint primarily permits flexion/extension motion whilst the C1-C2 human joint accounts for approximately 50% of the total axial rotation ROM of the cervical spine. The majority of this rotation is known to occur in the neutral zone, i.e. with low resistance to motion. The C0-C1 joint of the LUSN was therefore designed to permit the calculated ROM in flexion/extension and lateral flexion and the C1-C2 joint was limited to pure axial rotation and a ROM of 80° (40° to the left and right). The LUSN middle joint is similar to the C0-C1 upper joint mechanism but the high ROM was achieved using two mating truncated hemispheres (i.e., a ball with threaded joint), each accounting for half of the total ROM. The truncated hemispheres and bolt inserts are assembled in to the upper and lower segment bearing surfaces using threaded holes. The contours of the two bearing surfaces defined the ROM limit at the joint, whilst additional clearance was given to the truncation of the hemispheres to avoid interference of the bolts with the inner surfaces during maximal ROM. The LUSN lower joint design was an adaptation of the upper joint given the similarities in DOF and ROM. The slope of the first thoracic (T1) human vertebra was maintained on the T1 mechanical component and the flat surface acted as a ROM limit for the mating, contoured surface of the C7 component. The truncated hemispherical extrusion was constructed with its centre at the C7-T1 ICR location and the truncation allowed for maximal ROM and clearance to avoid interference of the bolt with the inner surface. Across each joint, a mixture of stainless steel and phosphor bronze were chosen to ensure low resistance at the joint. Figure 4 shows the human cervical spine CAD model and LUSN three-joint equivalent structure in combination with the biofidelic surrogate head of Miyazaki et al. (2017).

### Resistance to motion

The resistive elements were inspired by the cervical spine’s longitudinal ligaments, attaching anteriorly, posteriorly and laterally across each of the three LUSN joints. An analytical solution of the force required to displace the neck a known distance was used to inform the nominal material properties required for the passive response of the neck (i.e., cross-sectional area, length and Young’s modulus). Passive bending moment responses of human volunteers (20 male volunteers, stature: 1775mm; age: 19.5 ± 1.4 years) in flexion/extension and lateral flexion have been reported by McGill et al. (1994) and these values compare well to the cadaveric cervical spine response values of McElhaney et al. (1988). The bending stiffness of the human neck is known to be higher in flexion *versus* extension, though the difference in maximum bending stiffness was found to be relatively small (0.128 Nm/deg *versus* 0.106 Nm/deg) and similar to the lateral flexion stiffness of 0.13 Nm/deg. The values reported are a valid guide but must be interpreted with caution given the disparity in response reported both inter and intra respectively for human samples.

The geometry of the neutral posture was used to define the coordinates of the elastic element attachment locations, in order to determine the original length and perpendicular distance of the line of action of the resistive element with respect to the joint centre location. The total angular displacement of the head relative to the torso, as reported in McGill et al. (1994), was proportionally split across the three joints relative to their total possible ROM. The three joints were then analysed in isolation such that the respective angular displacement value was applied to the joint. The joint was considered to be in equlibrium at this moment in time as though the total moment, M , as reported in McGill et al. (1994) was applied to each joint. The 3D CAD (Siemens NX 8.5) model was then used to determine coordinate data for the new locations of the element attachments and Pythagoras’s theorem was used to calculate the elongation of the posterior and/or anterior elastic element. The force exerted by the anterior and posterior elements across each joint was then calculated for the respective bending mode.

The material stiffness characteristics of woven Polyester elastic (Stretchline Limited, width: 25mm, thickness: 3 mm) samples were quantified through tensile testing on an Instron Dynatup 9250HV machine. A nominal length between the loops was defined as 30 mm (approximately the length of the anterior middle joint elastic element). The loops were stitched to ensure that a standard sized (8 mm diameter) Instron testing pin could be used to secure the sample. A tensile loading profile was created such that an absolute ramp rate of 500 mm/min (maximum capacity of the machine) was applied until a load of 200 N was reached. The sample was then unloaded to its original length and the cycle was repeated 100 times. The sample was visually inspected after the loading protocol and the stitching was assessed for signs of damage. From the theoretical calculations, the maximum elongation of the elastic elements was predicted to be 43%, 38% and 22% at the top, middle and bottom joints, respectively. The stiffness modulus of the elastic sample was therefore predicted in the range of 0.2–0.6 mm/mm strain corresponding to a maximum of 18 mm elongation. An assessment of the stress *versus* strain gradient within this region resulted in a predicted modulus of 6.86 MPa, which is in agreement with the required modulus from the theoretical prediction.

**FIGURE 5:**
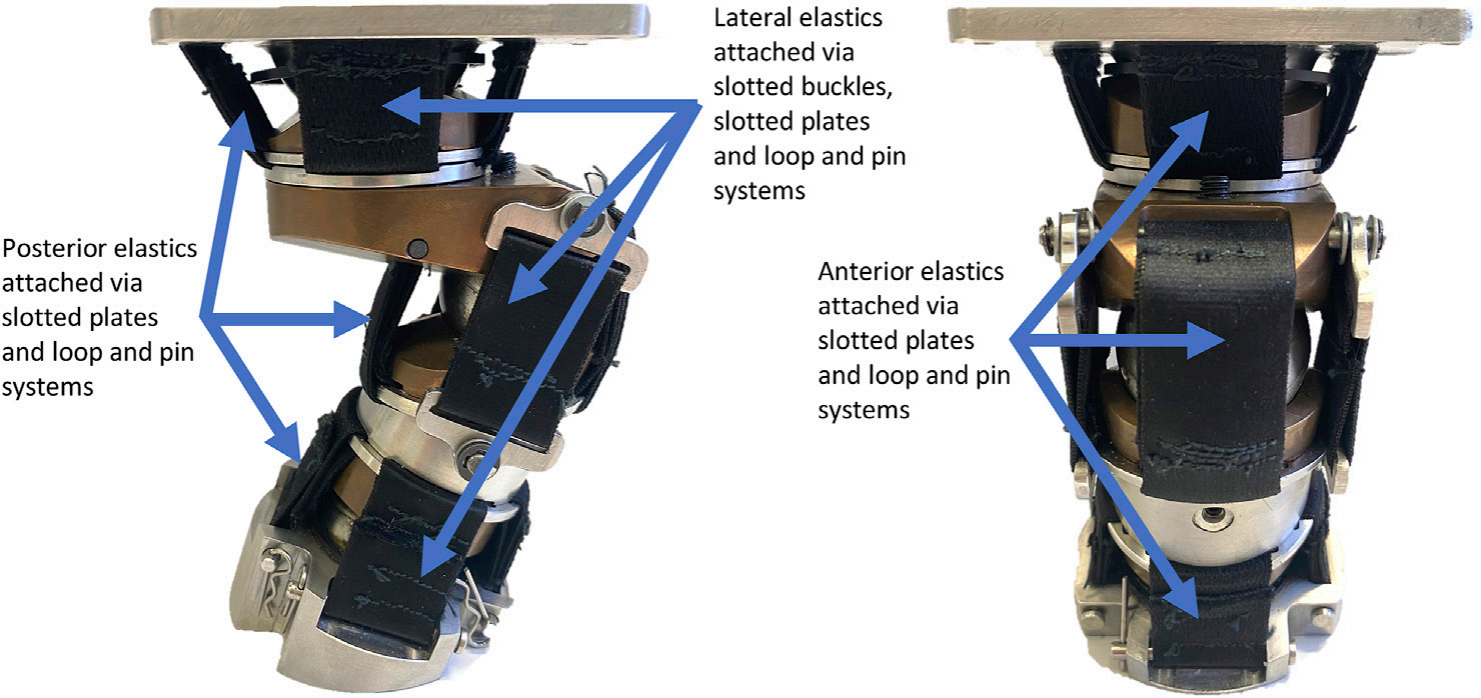
LUSN fully assembled in the neutral posture with elastic elements.

The Polyester elastic was cut into lengths defined by the CAD model and closed loops were formed at both ends with a bartack zigzag stitch. The elastic was either directly attached to a LUSN *via* a slotted plate and then stitched or attached retrospectively *via* a pin through the loop of the elastic. The configuration allows for lengths to be changed and therefore pretension of the elastic elements to be easily modified, which in turn can be used to define a non-neutral initial posture. The axial rotation plain bearing assembly is independent of the upper resistive elements and therefore is still able to rotate with a negligible resistance to motion, whilst the torsional stiffness of the lower neck elastics resist further axial rotation. Figure 5 shows the assembled neck in the neutral posture with elastic elements attached, along with annotations for clarity.

### Biofidelic response validation

The degree of biofidelity is defined as the ability of the LUSN to appropriately represent available data on the 50th percentile male (e.g., mass, range of motion and bending moment response). Specifically, the surrogate neck, when constrained at the lowest most component (representative of the human T1 vertebra) and subjected to a pure bending moment or axial torque at the upper most component (representative of the base of the skull, referred to as C0), should rotate nominally to an angle that is in agreement with that reported for the human in an equivalent load case. To quantify the biofidelity with respect to the definition, two separate experimental investigations were conducted, like those described in the literature (Luck et al., 2014).

### Inertial properties

The total mass of the surrogate neck considered all components except the T1 components (T1 body, T1 pins and T1 bolt insert). *In-situ* these components are rigidly mounted to the thorax and contribute 0.44 Kg of mass. Kang et al. (2016) reported mass and inertia of the upper (C0-C4) and lower (C4-C7) cervical spine. The equivalent upper and lower split on the LUSN is at the centre of the mid ball joint. A comparison of the total and upper and lower distributed mass is presented in the results. Estimates of the human cervical spine’s principal moments of inertia have rarely been reported, and those that have provide a wide range of values, due to differences in measurement methodologies and rigid body assumptions. For comparison, the theoretical inertial properties of the LUSN were calculated in the CAD software. The I_xx_, I_yy_ and I_zz,_ account for moments of inertia about the sagittal, coronal and transverse planes, respectively.

### Quasi static pure bending methodology

**FIGURE 6:**
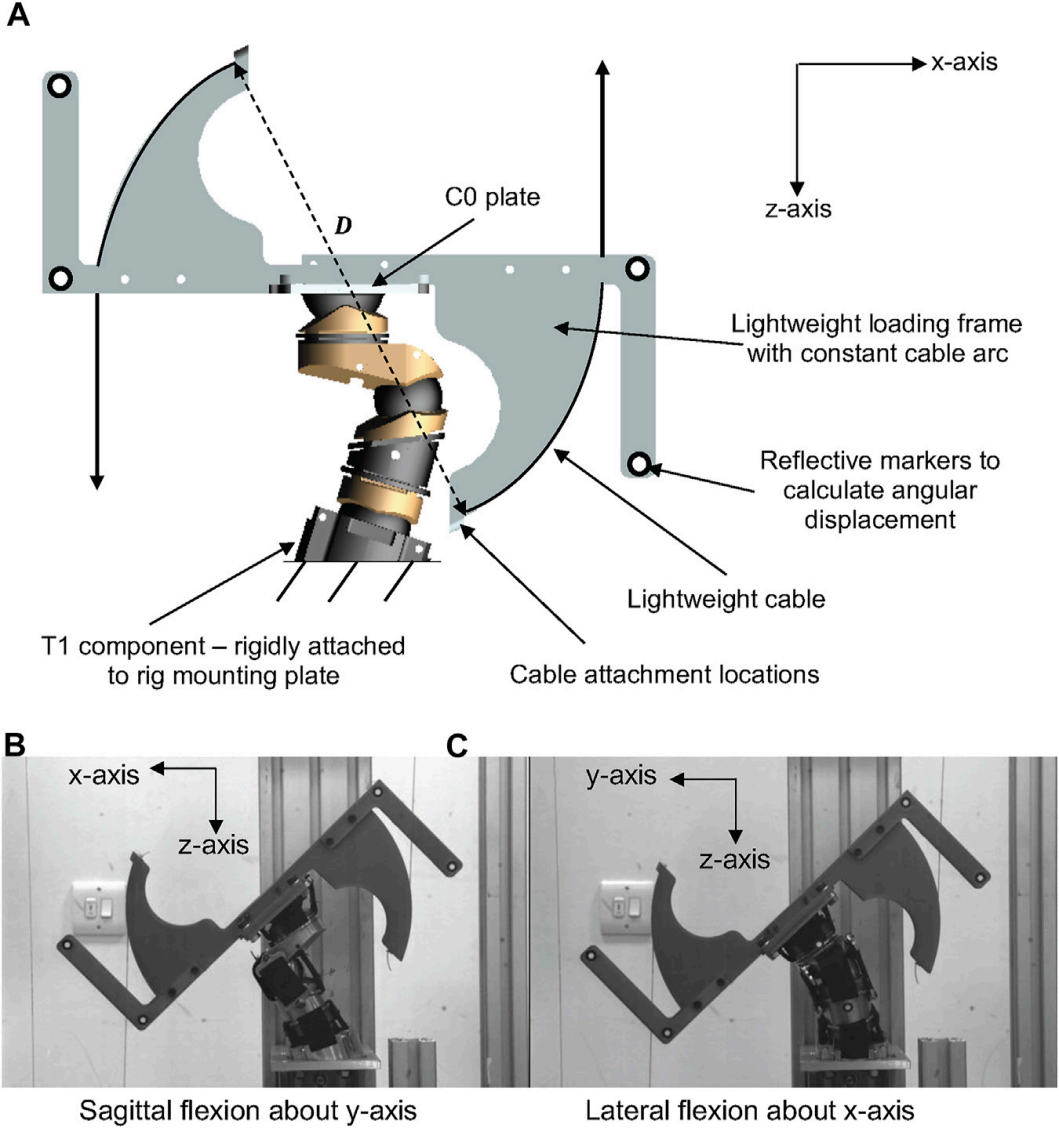
**(A)** Schematic representation of the LUSN in the neck bending rig. **(B)** Exemplar loading image of the LUSN in sagittal flexion about the *y*-axis. **(C)** Exemplar loading image of the LUSN in lateral flexion about the *x*-axis.

Based on the methodology adopted by Luck et al. (2014) and originally presented by Nightingale et al. (2002), the quasi static pure bending response of the LUSN was quantified. A loading frame was additively manufactured from ABS and rigidly mounted to the top of the C0 skull attachment plate of the LUSN. Two lengths of lightweight nylon cable were attached to the loading frame and masses were added at a constant arc guide of diameter 0.28 m. The cable attachments were located equidistance apart to ensure that equal and opposite forces were applied to the LUSN. The pulleys were lightweight with low inertial resistance and were positioned such that the vertical distance between the upper and lower pulleys were maximised to ensure that the force application resulted in rotation of the LUSN and minimised translation throughout the entire ROM. The bending rig mounting plate was designed to allow for the LUSN to be rotated 360° and rigidly mounted in four positions (90° increments), such that the application of the bending moment resulted in flexion and extension (about *y*-axis) and left and right lateral flexion (about *x*-axis). An annotated schematic of the setup is shown in Figure 6A.

In each of the bending orientations, the neutral posture of the surrogate neck was checked prior to load application and a calibrated digital protractor was used to ensure a consistent repositioning of the surrogate neck between trials. In the neutral posture, a camera (resolution 1024 x 640 pixels) was used to capture a calibration image of the LUSN after each repositioning, which would serve as reference images, from which the angular displacement of the neck would be calculated for each load case. The calibration images were captured with a 30 cm steel rule in view and this allowed for a pixel-to-mm calibration factor to be calculated. Additionally, retro-reflective circular markers were located on rigid arms of the lightweight loading frame to be tracked during post-processing.

Prior to testing, the LUSN was preconditioned with 10 cycles of 2.75 Nm applied bending moment. The masses (0.25 kg to 2.75 kg) were then added to each cable incrementally using a load, hold and unload approach, resulting in an applied moment of 0.69 Nm—7.55 Nm. After being secured to the cables, the masses were lowered slowly until the cable became taut and was then released from the hands of the tester. An image was captured 30 s after the application of the load to account for creep within the structure. The load was then removed, and the neck was placed back in the neutral posture before a calibration frame was captured and a subsequent load was applied. The full loading process was repeated five times in each of the principal loading orientations (about the x and y–axes) of the LUSN. Exemplar images of the sagittal and lateral loading cases are shown in Figures 6B,C, respectively.

Manual image processing was conducted using Image Pro Analyser software. The intra-tester repeatability of the testing was investigated first by quantifying the accuracy of the repositioning of the neutral posture of the LUSN and secondly by quantifying the accuracy of the digitisation of landmarks in the image analysis software. To investigate the repositioning of the LUSN, the landmarks of the lightweight fixture and features on the lower neck segments were digitised across each of the calibration images. A three-point circle feature was used to create a circle for each of the landmarks to quantify the coordinates of the circle’s centre location and the diameter of the circle. The accuracy of the landmark digitisation was investigated by selecting one calibration image from each bending mode and repeating the digitisation of four landmarks from each image, five times. The landmark data for both investigations were compared, and the mean and standard deviation values were calculated.

### Low-rate dynamic torque methodology

**FIGURE 7:**
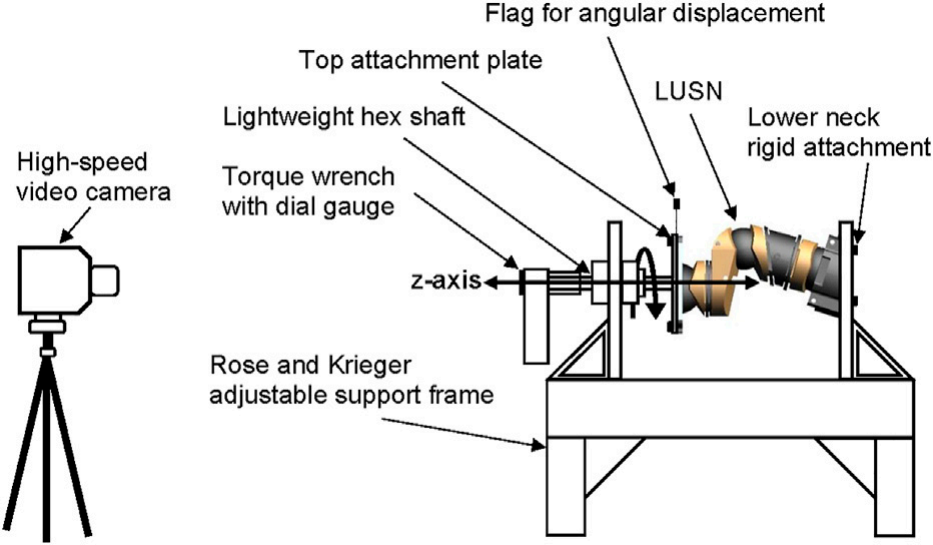
Schematic representation of the axial torque test setup.

A bespoke axial torque test rig (Figure 7) was developed to investigate the angular displacement response for axial torques applied about the *z*-axis. A simplified and manual bespoke test rig was constructed to quantify the axial torque response of the LUSN about the *z*-axis, based on the test setup used by Myers et al. (1989). A lightweight hex bar was attached to a rectangular top plate and bolted to the top of the LUSN, guided through a closed linear bearing. The sub-assembly was free to axially rotate and translate with negligible resistance to motion. The ability to axially translate was important due to the change in length of the neck during axial rotation. The constraint against this natural motion artificially increases the axial stiffness of the cervical spine (Myers et al., 1989). The torque was applied manually by the tester using a torque wrench (Kennedy Tools; model TW4) with a range of 0.3 Nm—4 Nm, a resolution of 0.1 Nm and accuracy of ±3% *via* the lightweight hex bar.

A low-speed loading rate of between 25 ( ± 5) degrees per second, was utilised for the battery of tests on the LUSN. The loading rate was equivalent to the loading procedure used by Luck et al. (2014) to quantify the characteristics of the Thor-M and Myers et al. (1989) to quantify the responses of PMHS. Furthermore, the H3SN data presented by Myers et al. (1989) was acquired with a loading rate of 30° per second. Therefore, the effect of loading rate on the response of the LUSN was minimised and ensured that a comparable dataset was collected. Prior to data collection, the LUSN was preconditioned with 10 cycles of axial rotation from 0 to 50°.

A high-speed video camera (Photron Fastcam SA1.1) recorded the application of the torque and was focused on the dial gauge of the torque wrench and also the lightweight, rigid markers on the upper neck attachment plate. The frame rate of the camera was 240 FPS to allow for analysis of the applied torque and angular displacement response. In addition, the applied loading rate was quantified by investigating the change in angular displacement of the torque wrench across frames of the high-speed video, ensuring that the torque was applied consistently. A total of ten trials were collected and analysed for the axial torque response.

### Surrogate head harmonic frequency response

The resonant frequencies of the human skull have previously been investigated using computational and experimental techniques to further the understanding of dynamic loading effects on the risk of head and brain injury. Ruan and Prasad (1996) used a finite element head model to investigate the effect of boundary conditions and skull thickness on the resonant frequency response of the head. Their results implied that by applying the boundary conditions at the lower neck instead of at the atlanto-occipital joint (upper most neck joint), the frequency content at each mode was reduced, with a significant effect at the lower modes (one to three). It is assumed that this would be due to an increase in the effective mass of the system. Khalil and Viano (1979) investigated the modal response of a single dry 50th percentile male cadaver skull, using a force hammer to excite the skull in several locations. 11 resonant frequencies were identified and the lowest was reported as 1385 Hz. Hakansson et al. (1994) conducted *in vivo* testing using human volunteers and found between 14 and 19 resonant frequencies for each of the six volunteers (2 males and 4 females). The lowest mean resonant frequency was 972 Hz and the standard deviation was ±119 Hz.

The dynamic loading of high-speed, short-duration impacts (e.g., baseball pitches) are likely to excite a wide range of frequencies. A biofidelic surrogate neck should not inhibit these responses before their influence on the resulting head response is understood. Therefore, the effect of surrogate neck constraint on the modal response of a biofidelic surrogate head (Miyazaki et al., 2017) was investigated. The modal response of the surrogate head was investigated under three constraints:

1) Freely suspended *via* lightweight bungee cords (typical constraint for laboratory high-speed projectile impact testing),
2) Constrained by the H3SN (most commonly utilised neck constraint in sport testing),
3) Constrained by the LUSN.

The surrogate head was fully assembled to contain all surrogate components with exception of the skin, which is known to dampen the frequency response of the skull and increase the difficulty of mounting an accelerometer. The head was constrained by one of the three stated conditions and a force hammer (Bruel and Kjaer, Type 8206-001) was used to apply an excitation load at locations around the surrogate head. The response was measured using a triaxial accelerometer (DJB Instruments, Type AT/10-6), mounted to the contralateral side of the surrogate head with synthetic wax and recorded using the Siemens LMS Test Lab 15.0. The excitation and measurement locations are summarised and visualised in Table 3.

**TABLE 3:** Definition of excitation and measurement locations used to investigate the resonant frequency response of the surrogate head when constrained by bungee cords, the LUSN and the H3SN.

Impact number	Excitation location	Measurement location	Excitation (E) and measurement M) locations
1	Left mid-side	Right mid-side	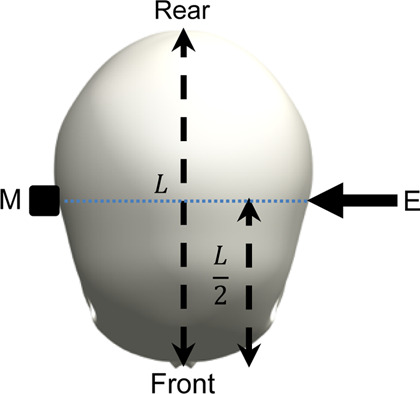
2	Left rear-side	Right mid-side	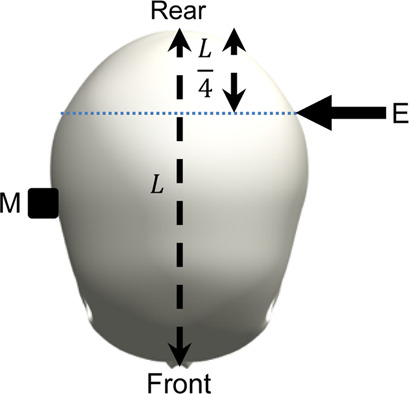
3	Left front-side	Right mid-side	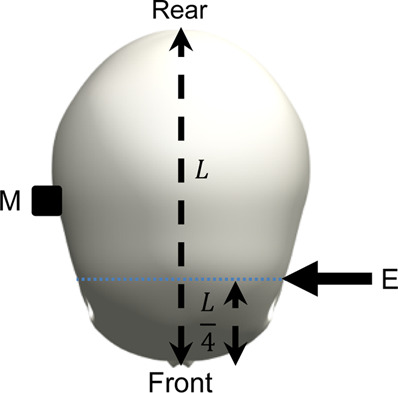
4	Frontal	Rear	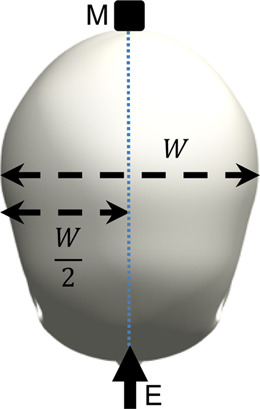
5	Rear	Frontal	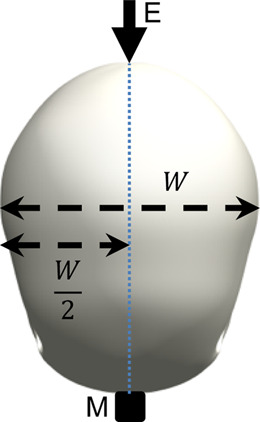

## Results

### Range, distribution and loci of motion

Table 4 presents the comparative percentage distribution of the total ROM across the joints of the LUSN and the equivalent summation across the human intervertebral joints (Waiter, 2006) in flexion/extension and axial rotation/lateral flexion, respectively. The LUSN and the cervical spine model were articulated through to their end ROM in flexion, extension and lateral flexion and the coordinates of the C0-C1 ICR were compared. The LUSN, using only three joints with distributed ROM, precisely matches the location at the end ROM and can closely approximate the predicted human loci of motion when considering the articulated human vertebral model. The maximum horizontal and vertical offset differences of the C0-C1 ICR were found to be 0.61 mm, 1.43 mm, 1.14 mm, and −0.31 mm, −0.20 mm, 0.05 mm for 50% ROM in flexion, extension and lateral flexion, respectively.

**TABLE 4:** The comparative percentage distribution of the total ROM across the joints of the LUSN and the equivalent summation across the human intervertebral joints in flexion, extension, axial rotation and lateral flexion.

Joint	Flexion		Extension	
	LUSN distribution (%)	Human distribution (%)^a^	LUSN distribution (%)	Human distribution (%)^a^
Top	29.26	36.37	40.91	36.37
Middle	51.66	50.86	44.38	50.86
Bottom	19.09	12.78	14.71	12.78

	Axial rotation		Lateral flexion	
Joint	LUSN distribution (%)	aHuman distribution (%)	LUSN distribution (%)	aHuman distribution (%)
Top	44.44	45.00	21.96	28.97
Middle	27.78	40.00	51.89	58.74
Bottom	27.78	15.00	26.15	12.28

^a^ Waiter (2006).

**FIGURE 8:**
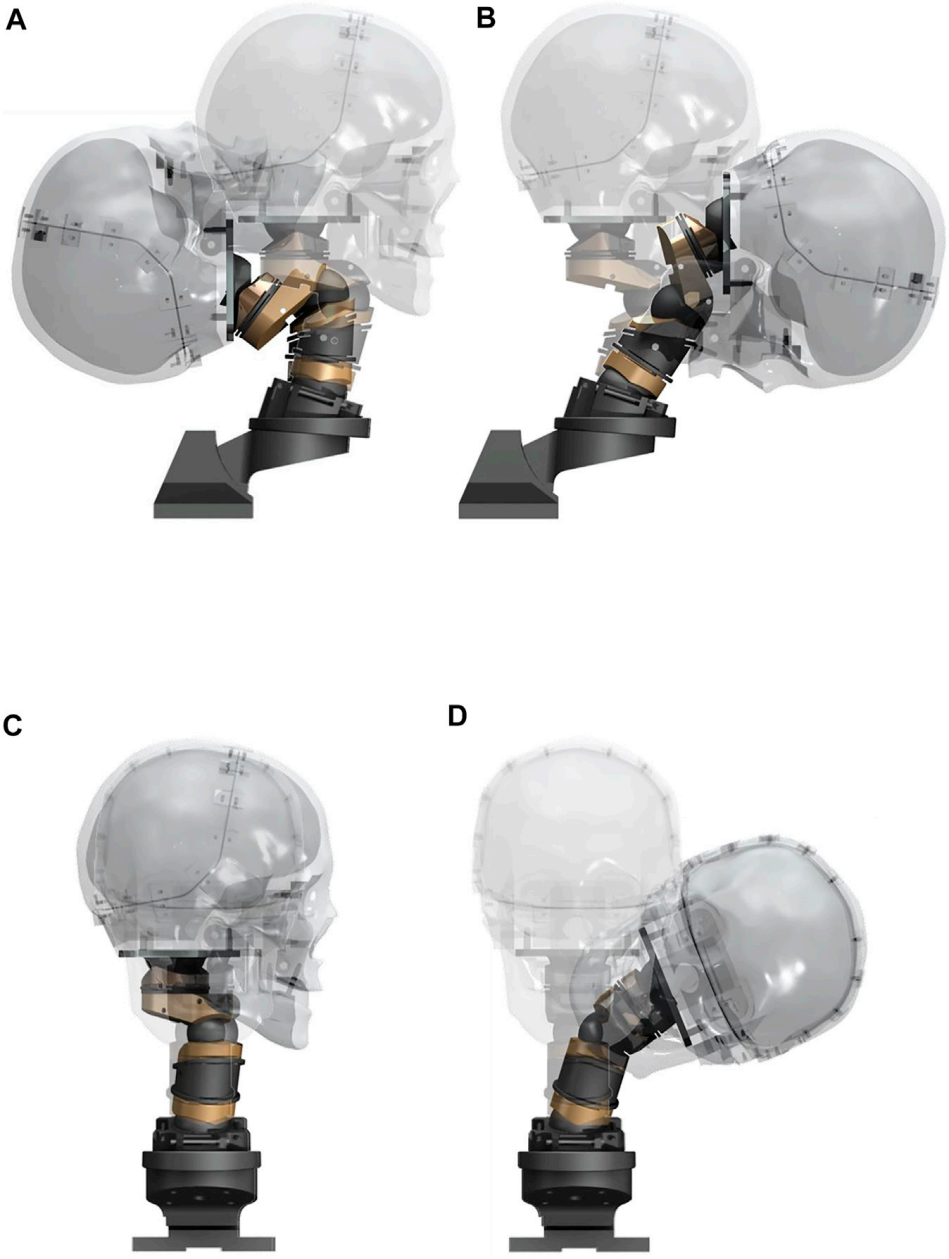
Rendered overlay images of the LUSN in full ROM with respect to the neutral posture during **(A)** Extension, **(B)** Flexion, **(C)** Axial rotation, **(D)** Lateral flexion.

Figure 8 presents rendered overlay images of the LUSN in full extent of ROM with respect to the neutral posture, in each of the principle planes.

### Inertial properties

The total mass of the LUSN is 1.62 Kg and has agreement with the total mass of the 50th percentile human cervical spine (1.6 Kg) (Kang et al., 2016). The total and distributed mass and moments of inertia, when considering an equivalent upper and lower cervical split, are presented in Table 5.

**TABLE 5:** Comparative (whole and distributed) mass and (whole) moments of inertia of the LUSN, H3SN and human neck a McConville and Churchill (1980), b Kang et al. (2016).

	Mass (kg)			Moments of inertia (kg.cm2)					
	LUSN	H3SN	Human	LUSN	Human	LUSN	Human	LUSN	Human
				Ixx		Iyy		Izz	
Upper neck	0.95		0.8						
Lower neck	0.67		0.8						
Whole neck	1.62	1.5	1.6	26.8	9.2–33.7a	20.5	10.2–36.8a	14.3	13.9–49.2a
					34.8–46.8b		29.2–41.7b		22.4–35.3b

### Bending moment and axial torque response corridors

**FIGURE 9:**
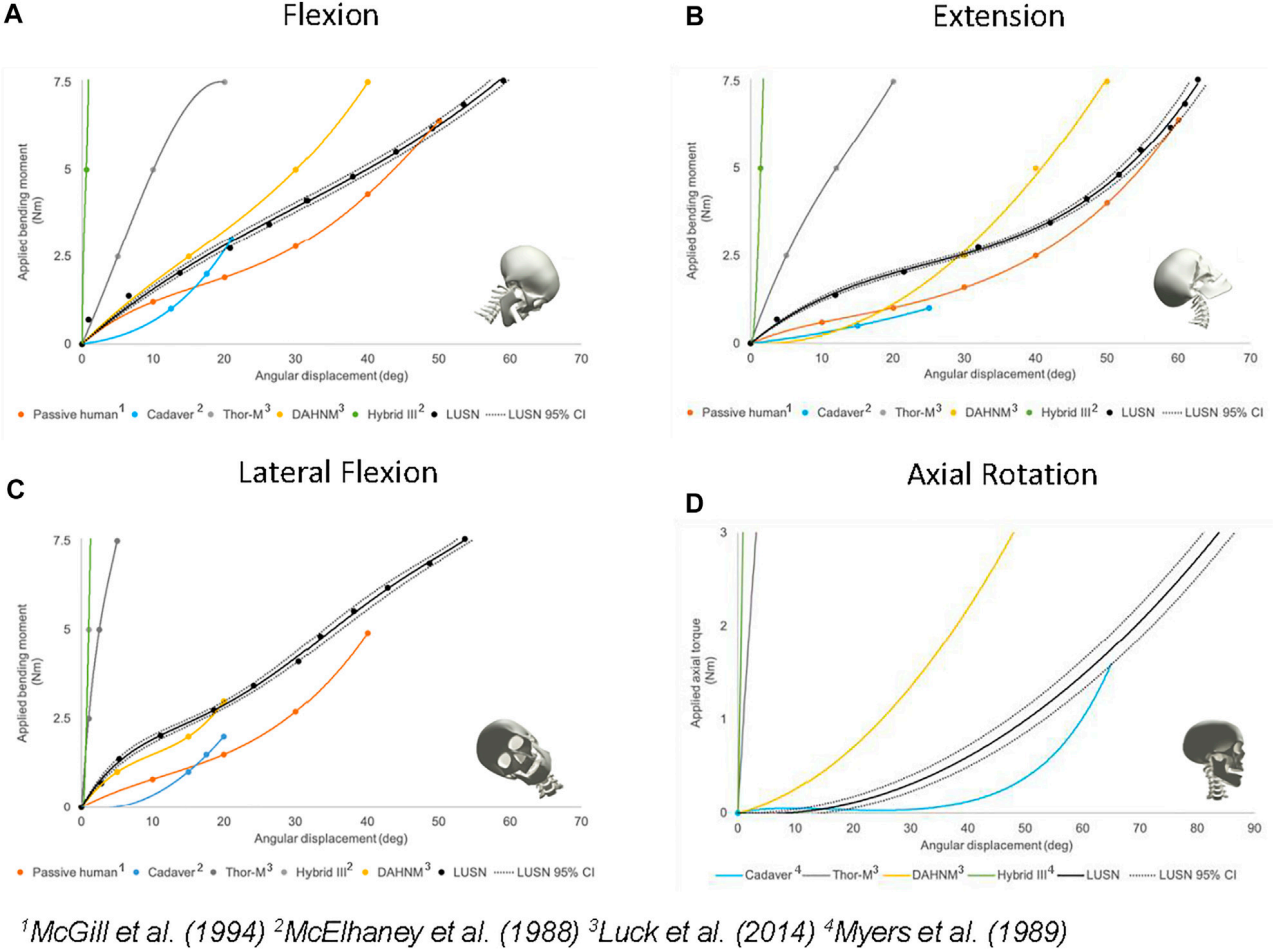
The validated moment or torque *versus* angle response corridors of the LUSN with respect to the best available human and surrogate data in: **(A)** Flexion bending **(B)** Extension bending **(C)** Lateral flexion bending **(D)** Axial rotation.

Figures 9A–C presents the mean angular displacement and 95% confidence intervals (CI) for each bending load case calculated for the LUSN. The results are presented as best-fit polynomial curves with an intercept of (0,0) and therefore represent an interpolation of the 11 measured angular displacements. For clarity, the 11 applied moments and corresponding angular displacements are presented as markers on the curves. The applied axial torque with resulting mean axial rotation and 95% CIs are presented in Figure 9D. Additional datasets (e.g., cadaver) have been interpreted as a series of x, y coordinates from those graphically presented by the respective authors and then interpolated using polynomial best-fit curves. Where possible, these results have been presented up to the maximum tested on the LUSN (7.5 Nm for bending modes and 3 Nm for axial rotation). The Thor-M surrogate neck and Duke Adult Head and Neck Model (DAHNM) response curves (bending and axial rotation) were interpreted from the quasi-static load cases with the necks in their no muscle cables and relaxed muscle states, respectively (Luck et al., 2014). The flexion, extension and lateral flexion H3SN and cadaveric data sets were interpreted from those presented by McElhaney et al. (1988) under tension-bending load cases. The axial rotation H3SN and cadaveric response curves were interpreted from the constant velocity tests of Myers et al. (1989). Additional response curves of human volunteers in the passive neck state are presented for the bending modes (McGill et al., 1994).

The bending moment response of the LUSN was found to be repeatable with a maximum deviation in angular displacement of ±0.77° across the applied loads. The higher loading cases generally resulted in a slightly higher measured deviation in angular displacement between the five repeats. The axial rotation response of the LUSN was also found to be highly repeatable across the range of applied axial torques. The maximum standard deviation (±1.95°) occurred at an applied torque of 2 Nm, where the range of axial rotation was 56–61°. Whilst the LUSN exhibits a highly repeatable response throughout the tested range, it is not possible to quantify this repeatability with respect to other surrogate necks, as the data has not been presented in the literature.

#### Flexion bending response

The typical flexion bending response of the LUSN (Figure 9A) showed a relatively linear relationship between applied bending moment and the resulting angular displacement throughout the range, with an increasing stiffness evident at the higher ROM values. The LUSN was initially stiffer than the cadaveric response (McElhaney et al., 1988) though showed agreement at 3 Nm of applied load (maximum reported for cadaver). With respect to the DAHNM computation model, between 0 Nm and 2 Nm of applied load, the LUSN has a similar angular displacement response. Beyond 2 Nm of applied load, the DAHNM model is stiffer than the LUSN. In contrast, the passive human data is less stiff than the LUSN throughout the entire ROM, though the original passive human data exhibited a high variation in the response between volunteers. A total angular displacement of approximately 59° was achieved for an applied bending moment of 7.55 Nm. The Thor-M response was stiffer than the LUSN throughout the tested ROM. In addition, the Thor-M was stiffer than the passive human, cadaver and DAHNM throughout its ROM. Interestingly, the stiffness of the Thor-M decreased with an increasing ROM. The H3SN, as expected, exhibited a high stiffness and linear relationship throughout the tested ROM.

#### Extension bending response

The general trend of the extension bending response for the LUSN (Figure 9B) was similar to the passive human, cadaver and DAHNM prediction responses, throughout the ROM. The neck stiffness response was reasonably linear up to approximately 40 degrees of angular displacement, beyond this region the neck stiffness increased with an increase in ROM. In the 0–30° angular displacement region, the LUSN is stiffer than the reported passive human and DAHNM model. Beyond this region, the difference in response decreases between LUSN and the passive human, whilst the DAHNM model appears to increase in neck stiffness, moving further away from the passive human response. A total angular displacement of approximately 63° was achieved for an applied bending moment of 7.55 Nm. The Thor-M exhibited similar characteristics to those in the flexion response and was stiffer than any of the other neck responses throughout its ROM, with the exception of the H3SN. Again, the Thor-M neck stiffness is greatest at the lowest values of ROM (0–5°) and then decreases before exhibiting a highly linear relationship between applied load and angular displacement. The H3SN has the highest neck stiffness and a linear response throughout the ROM.

#### Lateral flexion bending response

The general trend of the lateral flexion bending response for the LUSN (Figure 9C) was similar to the passive human and DAHNM prediction responses, with a stiffening response towards the higher ROM. The LUSN exhibited a similar response in left and right lateral flexion, owing to the symmetrical nature of the design about the mid-sagittal plane. At the low ROM region (0–20°) the LUSN has a slightly stiffer response than the DAHNM and passive human. Beyond this region, the LUSN is similar to the response of the DAHNM, though stiffer than the passive human. A total angular displacement of approximately 54° was achieved for an applied bending moment of 7.55 Nm. Similar to the flexion and extension behaviour, the Thor-M is stiffer than the LUSN, DAHNM and passive human throughout the ROM. Similarly, the Thor-M neck decreases in stiffness with an increasing angular displacement. The H3SN is the stiffest of all neck conditions and again, exhibits a linear response between applied load and resulting angular displacement.

#### Axial torque response

The general axial torque response characteristics of the LUSN (Figure 9D) show close agreement to the computational (DAHNM) predictions and cadaver (Myers et al., 1989) response curves. The LUSN is in agreement with the cadaver response in neutral zone (low resistance) region. Beyond this region, i.e. in the elastic zone, the LUSN is however, less stiff than the computational prediction of the DAHNM and stiffer than the cadaver. The LUSN rotated to approximately 85° with the application of 3 Nm of torque. In stark contrast, the Thor-M surrogate neck did not exhibit a low stiffness, high rotation response and was therefore not capable of representing the neutral zone of the human cervical spine in axial rotation. For reference, the Thor-M response presented in Luck et al. (2014) was stiffest during the first 10 degrees of axial rotation and had a softening response to approximately 40 degrees of rotation. Beyond this rotation, the surrogate exhibited a linear response and rotated to 80° with the application of approximately 35 Nm of torque. The H3SN had the highest torsional stiffness of all neck conditions and failed to rotate beyond approximately 2°.

#### Natural frequency response

**FIGURE 10:**
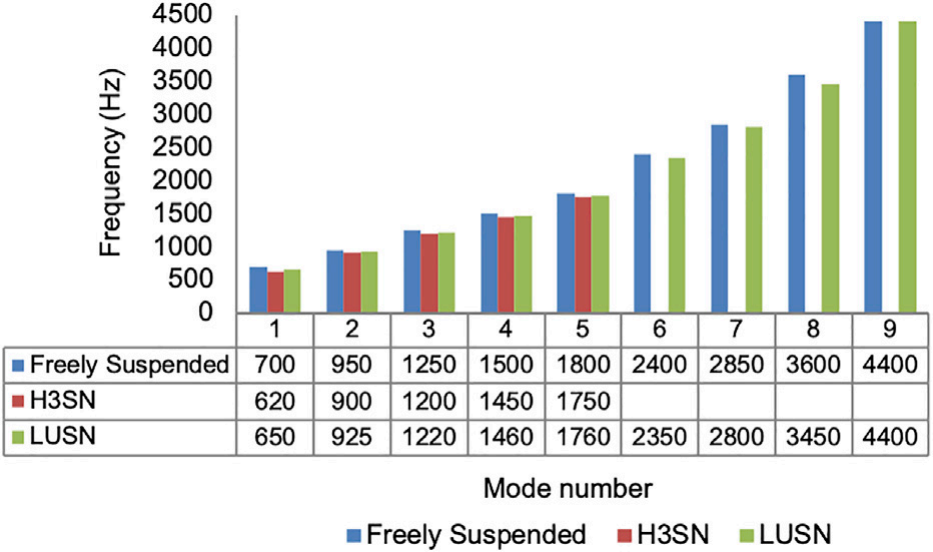
Modal response of the biofidelic surrogate head when constrained by bungee cords, the LUSN or the H3SN (Impact number 1, Table 8.3).

The measured frequency response of the surrogate head when constrained with the three different neck conditions and subjected to an excitation at the left mid-side location and measurement at the right mid-side location is presented in Figure 10. The results show up to nine resonant frequencies for the LUSN and freely suspended conditions and up to five for the H3SN condition. The minimum resonant frequencies were 700 Hz, 650 Hz and 620 Hz for the freely suspended, LUSN and H3SN conditions, respectively. The resonant frequencies generally decreased at each mode with an increase in neck constraint, i.e. the frequencies were highest for the freely suspended condition, reduced for the LUSN condition and reduced further for the H3SN condition. The H3SN tended to dampen the response of the surrogate head and the higher resonant frequencies that were typically seen on the LUSN and freely suspended conditions were absent when excited by these low-level impacts. The frequency response charts for the remaining impact locations can be seen in Supplementary Material S1.

## Discussion

A novel approach to the design and development of a surrogate neck was taken by considering the geometry of the cervical spine in a neutral lordotic posture. The digital model’s neutral posture was articulated using reported intervertebral ROM and ICR locations. This approach provided quantitative data on the intermediate and full ROM motion paths of the surrogate head, in each of the principal anatomical planes. The skeletal model predicted a lengthening and shortening of the neutral axial length of the spine in flexion and extension, respectively which influences the number and placement of joints that are necessary to represent the biomechanical response of the human neck. The model is advantageous in that other demographics (e.g., females) can be more appropriately represented by considering differences in their geometry, range of motion and instantaneous centres of rotation.

The encapsulated ball joints provide a high ROM in multiple directions and ensure that the upper and lower segments do not fall apart, even without the presence of resistive elements. This was desirable as it allowed for the timely development of a bracing structure, without the need for it to constrain the joint from the outset. Further advantages of the joint solution are that the tension provided by the bolts in to the lower and upper segments, when torqued appropriately, prevent rattle which is inherent in joints that are reliant on the bracing structures. The design prevents alignment issues which can result from rattle or ‘stretching’ across the joint and with inference, the design increases the repeatability of the response due to the constraint. Furthermore, the independence of the joints to the resistive elements enables the head to be configured in any orientation within the stated maximal ROM. The non-neutral postures can be construed through simply changing the lengths of the individual elastic elements. This is likely to be advantageous for current and future test scenarios in sport and other areas (e.g., automotive), where participants and occupants are positioned in a non-neutral posture prior to impact. The limitation of the current solution, with respect to the human response, is that the neck will only partially axially compress and not in the manner expected from the complex intervertebral joints. The lordotic profile of the ATD neck will result in a natural axial buckling response, though the local stiffness is higher than that expected from the human cervical spine. Whilst the priority of many common sports related head injuries is not the axial stretch or compression of the neck, further work is necessary to evaluate the axial compression response of the current design. It is anticipated that modifications may be necessary to increase the axial compliance of the ATD neck when considering axial impact load cases such as head-first tackling in contact sports.

The total ROM of the LUSN is in agreement with the human data summarised by Waiter (2006). Three encapsulated ball joints of appropriate positioning and distribution of ROM were found to precisely position and orientate the head’s EAM and Frankfort plane at the end ROM. The maximum deviation of the head’s location due to the LUSN’s motion *versus* the human model’s prediction, during 50% ROM, was found to be 1.4 mm and 0.3 mm in the horizontal and vertical axes, respectively. The complexity of the eight intervertebral human joints was therefore reduced to three ball joints, whilst maintaining the high ROM in and between the anatomical planes. Furthermore, the importance of the human neck to axially rotate with a large neutral zone (accounting for approximately 50% of total axial rotation), has been addressed with the addition of a plain bearing sub-assembly at the C1-C2 vertebral region. The distribution of total ROM across the three LUSN joints was found to have agreement with the equivalent summated intervertebral ROM of the human neck, for example where the upper LUSN joint represents the C0-C1 and C1-C2 human joints. Whilst the LUSN does not necessarily reach the final ROM locations with the complex distributed approach modelled by the human vertebrae, the surrogate neck was designed to allow the head model to reach the end orientation and location precisely. It is acknowledged that increasing the number of joints would enable further segmental accuracy in the predicted human motion, however, it was not desirable due to the perceived decrease in stability of the head during a passive (i.e., low resistance) neck state. The increase in number of joints would also increase the complexity and cost of the surrogate and would likely decrease the repeatability of the positioning of the head before, during and after impacts.

The moments of inertia of the LUSN are in agreement with the human values reported by McConville and Churchill (1980). However, they are lower than the values reported by Kang et al. (2016). The human inertial values should be interpreted with caution due to the differences in segmental definition of the cervical spine and the assumption of a constant density in the approximation of the human neck. The addition of actively braced elements will increase the moments of inertia of the LUSN; however, it is not anticipated that these will lead to full agreement with the values of Kang et al. (2016). With respect to the reported values of the H3SN, the I_xx_ values are almost identical, whilst the I_yy_ and I_zz_ are smaller and larger, respectively.

The use of elastomeric elements (inspired by the anatomy of the cervical ligaments), provided a close and repeatable approximation of the resistance to motion response expected from the passive state human neck. The pretension length of the elastic elements was found to be an important determinant in the stiffening response of the LUSN and this was particularly evident in the flexion response which did not stiffen at the desired rate during the higher load application. The summative effect of establishing performance goals and successfully achieving them on the fabricated LUSN, is evident in the validation test results that have been presented. The response characteristics of the LUSN in each bending mode were found to be in agreement with the response curves reported in the literature. The response characteristics of the LUSN during axial rotation were also found to be in agreement with the response curves reported in the literature. The responses were consistent with low standard deviations throughout the neutral and elastic zones. However, repeatability of the surrogate manufacture and assembly needs to be quantified through testing additional prototypes. The human responses (cadaver and passive human volunteer) are known to be highly variable and it is therefore reasonable to infer that the LUSN will offer an improved repeatability compared to these surrogates or volunteers.

The responses were evaluated with respect to reported data on the Thor-M surrogate neck (best available technology) and the H3SN (most commonly used). The Thor-M consistently performed with its stiffest response at the lowest ROM and then decreased in stiffness. The general shape of this response did not agree with the human data (i.e. a low to high stiffness response with increasing ROM). In addition, the magnitude of angular displacement for the Thor-M was generally three times lower than that of the human and LUSN for any given applied load. A particular success of the LUSN, with respect to the best available technology can be seen in the axial rotation response characteristics. The LUSN exhibits a clear neutral zone which is attributed to the design of the plain bearing assembly, representing the C1-C2 vertebral interaction of the human cervical spine. The phase, shape and magnitude of the axial rotation response is in agreement with the human data throughout the entire ROM.

Whilst the comparison to the H3SN is important to the research community, it must be interpreted with caution. The design intent of the H3SN was not to represent the passive state human, rather it was designed to represent the tensed musculature of the human for a frontal automotive impact scenario. It could therefore be deemed unfair to assess the H3SN in this regard; however, the comparison is valid for the current research given the tendency for researchers to use the surrogate neck in TBI-related research. In particular, for impacts where a worst-case scenario may involve an “unbraced and unaware” participant. The results demonstrate that the response of the H3SN should be reserved for impact scenarios limited to the sagittal plane (flexion and extension motion) and for representing tensed neck musculature. The ability of the neck to laterally flex is limited and the axial rotation response of the H3SN showed the disparity with respect to the reported human and computational data (achieving approximately 3° for an applied torque of 9 Nm).

The dynamic response of the LUSN in each bending mode has not been quantified in this research and is required to enable a complete evaluation of biofidelity with respect to available human data and for comparison of performance with the alternative surrogate necks. Parent et al. (2017) presented comparative dynamic load response data for the Thor-M and H3SN and found the Thor-M to have a marked improvement in biofidelity during dynamic flexion/extension and lateral flexion modes. The dynamic dataset would also allow the LUSN to be compared to the human cadaver specimens and H3SN datasets (McElhaney et al., 1988), where the H3SN was found to be significantly affected by the applied strain rate and the human cadaver response was largely independent of strain rate. In addition, whilst the current elastic elements can be fine-tuned to increase or decrease the neck stiffness response, future development should focus on the active neck musculature response to enable a tunable LUSN which is suitable for the full spectrum of neck muscle activation responses.

With respect to the natural frequency testing of the biofidelic surrogate head, the LUSN was found to preserve the higher order frequency modes when excited by a low-level impact. The response was most similar to those recorded for a freely suspended constraint than the overly stiff H3SN. In support of the literature provided in the methodology, the results indicate that the neck constraint can influence the natural frequency response of the head. The results are particularly important when considering surrogate neck selection for highly dynamic scenarios such as projectile sports and ballistic impacts that are likely to elicit higher frequency responses of the head. It is therefore important that an appropriate neck condition is utilised that does not inhibit these responses.

To conclude, the LUSN is a prototype surrogate neck that constitutes four rigid segments with three ball joints to permit a distributed ROM, matching that of the 50th percentile male human. A fourth, single degree-of-freedom, plain bearing joint was added at the C1-C2 vertebral level to permit left and right axial rotation with negligible resistance. The LUSN is constrained by elastomeric elements which provide unbraced passive state resistance to motion across each joint and can be configured to enable non-neutral postures for impact tests. Initial validation tests showed the LUSN to perform similarly to the reported data and, in comparison to commercially available surrogate necks showed closer agreement to the human data in each plane of motion. However, further validation tests are required to characterise the LUSN’s responses under dynamic load cases and with respect to more clearly defined human response corridors. The LUSN can usefully be adopted into test scenarios as it has full compatibility with the commonly used H3SN surrogate head and torso.

## Data Availability

The raw data supporting the conclusion of this article will be made available by the authors, without undue reservation.
