# Supplementary material for: A human surrogate neck for traumatic brain injury research
Source: Front Bioeng Biotechnol. 2022 Dec 19;10:854405. doi: 10.3389/fbioe.2022.854405 (PMC9806148; doi:10.3389/fbioe.2022.854405)
Supplement: Supplementary file 1 [file DataSheet1.docx]

**Surrogate head frequency response (impact location numbers two-five)**

Figure 1- Impact location number two

Figure 2 - Impact location number three

Figure 3 - Impact location number four

Figure 4 - Impact location number five
